
JOURNAL INFORMATION
==============================
NLM Title Abbreviation: Wirtschaftsdienst
Journal ID: Wirtschaftsdienst
NLM Journal ID: 101086612

Wirtschaftsdienst (Hamburg, Germany : 1949)

ISSN: 0043-6275

ARTICLE INFORMATION
==============================
PMCID: PMC7335926
PMID: 32834153
DOI: 10.1007/s10273-020-2669-4
Article ID: 2669
Article version: 1
Subjects: Zeitgespräch

Ausnahmeföderalismus als Dauerzustand

Enderlein Henrik enderlein@hertie-school.org

Guttenberg Lucas guttenberg@delorscentre.eu

grid.424677.4 0000 0004 0548 4745 Jacques Delors Centre, Hertie School of Governance, Friedrichstraße 194, 10117 Berlin, Deutschland
Electronic publication date: 2020 Jul 6
Print publication date: 2020
Volume: 100
Issue: 6
First page: 400
Last page: 404
Copyright: © Der/die Autor(en) 2020
License: Open Access Dieser Artikel wird unter der Creative Commons Namensnennung 4.0 International Lizenz veröffentlicht, welche die Nutzung, Vervielfältigung, Bearbeitung, Verbreitung und Wiedergabe in jeglichem Medium und Format erlaubt, sofern Sie den/die ursprünglichen Autor(en) und die Quelle ordnungsgemäß nennen, einen Link zur Creative Commons Lizenz beifügen und angeben, ob Änderungen vorgenommen wurden.
Die in diesem Artikel enthaltenen Bilder und sonstiges Drittmaterial unterliegen ebenfalls der genannten Creative Commons Lizenz, sofern sich aus der Abbildungslegende nichts anderes ergibt. Sofern das betreffende Material nicht unter der genannten Creative Commons Lizenz steht und die betreffende Handlung nicht nach gesetzlichen Vorschriften erlaubt ist, ist für die oben aufgeführten Weiterverwendungen des Materials die Einwilligung des jeweiligen Rechteinhabers einzuholen.
Weitere Details zur Lizenz entnehmen Sie bitte der Lizenzinformation auf http://creativecommons.org/licenses/by/4.0/deed.de.
License URL: https://creativecommons.org/licenses/by/4.0/

issue-copyright-statement: © The Author(s) 2020

==============================

Mit ihrem Vorschlag zum EU-Wiederaufbaufonds haben der französische Präsident Emmanuel Macron und Bundeskanzlerin Angela Merkel am 18. Mai 2020 einen sehr wichtigen Schritt der Europäischen Union hin zu einer gemeinsamen Finanzpolitik auf den Weg gebracht. Das deutsch-französische Bekenntnis zur Möglichkeit der Europäische Union, Schulden aufzunehmen, ohne sie direkt und explizit über Kredite an Nationalstaaten abzusichern, sondern damit direkte Ausgaben in den Mitgliedsländern zu finanzieren, kommt einem kleinen Systembruch gleich. Man kann darin sogar in indirekter Analogie an die Geschichte der Vereinigten Staaten von Amerika eine Art

„Hamilton-Moment“ sehen, bei dem sich eine neue föderale Ebene dadurch konstituiert, dass sie nicht nur Schulden aufnehmen kann, sondern damit auch größere ausgabenseitige Autonomie erhält.[1]

Das Vorzeichen für diesen Schritt zu einer gemeinsamen Haushaltspolitik für Europa war eine Pandemie, die als symmetrischer Schock mit asymmetrischen Auswirkungen in den einzelnen Mitgliedsländern nach einer gemeinsamen Antwort verlangte. Dass diese Antwort nach längerem Zögern auf allen Ebenen so deutlich auszufallen scheint (aktuell ist der Macron-Merkel-Vorschlag zwar durch einen Umsetzungsvorschlag der Europäischen Kommission weiter konkretisiert worden, vom Europäischen Rat aber noch nicht verabschiedet), ist allerdings kein Bruch mit dem modus operandi der Eurozone, sondern die Fortsetzung einer Funktionslogik, die den Euroraum zu konstituieren scheint.

Die Krisenantwort der EU auf die Pandemie reiht sich nahtlos in eine Logik ein, die als „Ausnahmeföderalismus“, „Notfallföderalismus“ oder „federalism by exception“ beschrieben werden kann. Diese Tendenz, supra-nationales Handeln nicht zum Regelfall werden zu lassen, sondern es auf besondere Ausnahmesituationen zu beschränken und dann mit hohen Hürden zu versehen, ist zum wichtigsten politischen Modus im Euroraum geworden. Es ist zwar eine Stärke der EU, mit föderalen Ad-hoc-Antworten auf Krisensituationen zu reagieren, dies beantwortet aber gleichzeitig nur begrenzt die sehr grundsätzliche Frage nach der Reichweite politischer und ökonomischer Integration. Dies zeigen insbesondere die politischen aber auch juristischen Debatten immer wieder. Die EU (und vor allem die Eurozone) wird zwar mit jeder Krise stärker, sie wird ihre inhärente Instabilität aber auf absehbare Zeit nicht ablegen, weil auf Krisenzeiten begrenzte föderale Elemente keine ausreichende langfristige Stabilität herstellen können.

## Die Logik des Ausnahmeföderalismus

Im Maastricht-Vertrag wurde eine Wirtschafts- und Währungsunion in Europa verankert, die sich letztlich auf eine reine Währungsunion beschränkte. Von vollständiger wirtschaftlicher Integration konnte nie die Rede sein. Bundeskanzlerin Angela Merkel hat in mehreren Reden darauf hingewiesen: Viele Schwächen der Währungsunion sind auch darauf zurückzuführen, dass die Mahnung von Jacques Delors, beide Hälften des Projekts umzusetzen, nie ernst genommen wurde.[2]

Schon im ersten Jahrzehnt des Euroraums war damit ein wirtschafts-, finanz- und währungspolitischer Kontext angelegt, der permanent mit zwei zentralen Schwächen umgehen musste: einerseits mit dem Trend zu zyklischer Divergenz zwischen den Euro-Mitgliedsländern, ausgelöst durch die „one-size-fits-none“-Geldpolitik der EZB und durch divergierende Präferenzen und Herausforderungen in der Wirtschaftspolitik; andererseits durch die fast vollständige Abwesenheit von echten Krisenbekämpfungsmechanismen und echten haushaltspolitischen Instrumenten jenseits des EU-Haushalts (Enderlein et al., 2013).

Angesichts dieser Ausgangslage gab es von der Geburt des Euroraums an zwei große Lager in der Währungsunion: Das eine Lager setzte auf die Logik, dass jeder sein eigenes Haus in Ordnung halten müsse, weil Krisen dann gar nicht erst aufkommen würden; das andere Lager hielt dieses Vorgehen für eine Wunschvorstellung und drängte auf stärkere föderale Elemente. Spätestens zu Beginn der Krise im Euroraum zwischen Herbst 2009 und Frühjahr 2010 prallten die beiden Logiken mit aller Härte aufeinander. Während die eine Seite an der Argumentation festhielt, dass die Beibehaltung nationaler wirtschafts- und haushaltspolitischer Entscheidungsautonomie auch die Übernahme aller mit ihr verbundenen Konsequenzen nach sich ziehen musste, um „Moral hazard“ zu vermeiden, verwies die andere Seite auf das Risiko für den Euroraum in seiner Gesamtheit, sollte das Fehlverhalten einzelner Staaten nicht durch eine gemeinschaftliche Antwort korrigiert werden.

1 Wobei der Hamilton-Vergleich definitiv nur als verkürzte, wenn auch sehr starke Metapher taugt. Ausgangspunkt und Konsequenzen des Hamilton-Moments in den USA sind zu unterschiedlich für einen echten Vergleich.

2 Zum Beispiel in der Regierungsbefragung am 13. Mai 2020: „Wir dürfen nie vergessen, dass Jacques Delors vor Einführung des Euro gesagt hat: Es bedarf auch einer politischen Union; allein eine Währungsunion wird nicht reichen. – Wir sind da einige Schritte vorangekommen, aber wir sind nicht ausreichend vorangekommen. Das ist vollkommen evident. Das heißt also, es wird eher mehr Integration geben müssen als weniger, ohne dass ich heute hier schon spezifisch etwas sagen kann.“

Die gemeinsame Antwort des Euroraums war letztlich der Schritt zum Ausnahmeföderalismus: Mit dem sukzessiven Aufbau des Europäischen Stabilitätsmechanismus (ESM) wurde eine Funktionslogik vereinbart, in der Mitgliedsländer in einer Krise per Vertrag (Memorandum of Understanding, MoU) faktisch zu einer Abgabe ihrer wirtschafts- und haushaltspolitischen Autonomie gezwungen werden und im Gegenzug subventionierte Kredite der anderen Euroländer erhalten. Dabei bleibt in normalen Zeiten die nationale Wirtschafts- und Haushaltspolitik strikt in der Verantwortung der Mitgliedstaaten. So entsteht eine Fiktion zweier diskreter, klar voneinander unterscheidbarer Modi – Krisenland und Nichtkrisenland.

Die Europäische Zentralbank führte die Logik des Ausnahmeföderalismus unter Mario Draghi 2012 dann noch einen Schritt weiter, als sie die Föderalisierung der Krisenpolitik durch ein geldpolitisches Instrument erweiterte, die Outright Monetary Transactions (OMT), das auf Grundlage eines ESM-Programms die selektive geldpolitische Intervention zur Aufrechterhaltung des geldpolitischen Transmissionsmechanismus ermöglichte. Im Nachgang der Krise im Euroraum wurde oft argumentiert, dass die Antworten des Euroraums und der Europäischen Union keineswegs ausreichend sein würden, um eine neuerliche Krisensituation unter Kontrolle zu bringen und vor allem um Krisen effektiver vorzubeugen (vgl. z.B. Enderlein et al., 2012; Dawson et al., 2015; Enderlein et al., 2016; Bénassy-Quéré et al., 2018; Guttenberg und Hemker, 2018).

Mit der Corona-Pandemie entstand ein Krisenkontext, der die Europäische Union und vor allem die Eurozone mit den Unzulänglichkeiten ihres eigenen Instrumentariums zur Bekämpfung von Krisen konfrontierte. Schnell wurde deutlich, dass der Nexus zwischen einem hart konditionierten ESM-Programm und erst dann einsetzender länderspezifischer Stabilisierung durch die EZB für die Corona-Krisensituation völlig unangemessen war. In letzter Konsequenz führte die Pandemie die Krisenland/Nichtkrisenland-Fiktion ad absurdum: Wenn alle Länder in einer Krise sind, können nicht alle gleichzeitig Souveränität abgeben. Stattdessen stellte sich die Situation nun als ein Kontinuum unterschiedlicher Betroffenheiten dar, für die es differenzierte Antworten brauchte.

Entsprechend fielen gleich mehrere Tabus im Euroraum – allerdings immer versehen mit dem Hinweis, dass es sich um die strikt temporäre Einführung von neuen Instrumenten föderalen Charakters handelte. Am 18. März 2020 kündigte die EZB ihr „Pandemic Emergency Purchase Programm (PEPP)“ als geldpolitische Sondermaßnahme an. Mit dem PEPP legte die EZB ein Programm auf, bei dem sie ähnlich flexible und länderspezifische Interventionen wie im OMT auch ohne ESM-Programm, also ohne Aufgabe nationaler wirtschaftspolitischer Autonomie, umsetzen konnte. „Gleichzeitig werden Ankäufe im PEPP auf flexible Art und Weise erfolgen, um der außerordentlichen Lage in Folge der COVID-19 Verbreitung gerecht zu werden. Dies kann dazu führen, dass die Verteilung von Ankaufvolumen über die Zeit und Anleiheklassen hinweg, sowie zwischen Jurisdiktionen schwanken wird, um funktionsfähige Marktmechanismen zu unterstützen und eine reibungslose geldpolitische Transmission zu gewährleisten.“ (Deutsche Bundesbank, 2020). Geldpolitisch wurde mit dem PEPP eine klar föderale Komponente geschaffen, die allerdings wieder einer reinen Ausnahmesituation geschuldet und auf die Ausnahmesituation begrenzt ist.

Auch die am 18. Mai 2020 von Deutschland und Frankreich vorgeschlagene Funktionslogik eines „Recovery Funds“ folgt der Logik des Ausnahmeföderalismus. Die Verknüpfung der im Vertrag vorgesehenen Solidarität der Mitgliedstaaten untereinander in Krisenzeiten (Art. 122 des Vertrags über die Arbeitsweise der Europäischen Union, AEUV) mit der Feststellung, dass die EU sich gemäß Art. 311 AEUV in Ausnahmesituationen für diese Art von Solidarität auch durch Nettokreditaufnahme die notwendigen Finanzmittel zur Verfügung stellen kann, schafft ein eindeutig föderales haushaltspolitisches Element – allerdings wiederum strikt beschränkt auf Krisenzeiten.[3]

In der Pandemie haben sich Europäische Union, der Euroraum und ihre Mitgliedsländer also deutlich in Richtung stärkerer föderaler Elemente in der Geld-, Wirtschafts- und Haushaltspolitik bewegt, dabei aber lediglich einen weiteren Ausbau des Ausnahmeföderalismus befördert und keine dauerhaften föderalen Elemente aufgebaut.

## Wie dauerhaft ist der Ausnahmeföderalismus?

Dieses Vorgehen wirft die Frage auf, in welche Richtung sich vor allem die Wirtschafts- und Währungsunion im Anschluss an die Pandemie entwickeln wird. Die nun wieder bewiesene Handlungsfähigkeit der EU, mit föderalen Ad-hoc-Antworten auf Krisensituationen zu reagieren, beantwortet nur begrenzt die sehr grundsätzliche Frage nach der notwendigen und sinnvolle Reichweite politischer und ökonomischer Integration, wie insbesondere die politischen aber auch juristischen Debatten immer wieder zeigen (Enderlein und Guttenberg, 2019).

Allein die Schwierigkeit der Abgrenzung zwischen „Normalzustand“ und „Krisenzustand“ und wie oben beschrieben zwischen Krisen- und Nichtkrisenländern macht es schwer, oder sogar unmöglich, klar abzugrenzen, wann föderales

3 Zu den rechtlichen und ökonomischen Grundlagen des Instruments vgl. Grund et al. (2020).

Handeln angemessen ist – und wann nicht. Die Frage wäre auch rein rhetorischer Natur, wenn der politische Konsens für ein vollständig föderales System überall in der EU und vor allem im Euroraum vorhanden wäre. Da dies aber nicht der Fall ist, wird jede Einführung zusätzlicher föderaler Elemente zu einem politischen Testfall oder sogar einer juristischen Grundsatzdebatte über die Beibehaltung nationaler wirtschaftspolitischer Autonomie in der Europäischen Währungsunion. Solche Debatten sind sowohl in Ländern wie Griechenland, Irland oder Portugal zu Zeiten der MoU geführt worden, als die wirtschaftspolitische Autonomie dieser Länder durch das MoU durch eine quasi föderalen Instanz ersetzt wurde, wie auch in Deutschland gegenüber der EZB und der angeblichen Auswirkungen ihrer Geldpolitik auf die Sparzinsen oder Immobilienpreise. In beiden Fällen dient die im Maastricht-Vertrag vereinbarte Nicht-Vergemeinschaftung der Wirtschaftspolitik als Schlüsselargument der Kritik, die von einem vermeintlich ausufernden Föderalismus ausgeht.

Hier kann keine detaillierte Auseinandersetzung mit dem Urteil des Bundesverfassungsgerichts vom 5. Mai 2020 vorgenommen werden. Aber stark vereinfacht scheint das Gericht ein großes Unbehagen über föderales Handeln der Geldpolitik in denjenigen Bereichen der Wirtschaftspolitik zu treiben, die nach Auffassung des Bundesverfassungsgerichts ja gerade nicht vergemeinschaftet sind. Dieses Unbehagen zieht sich durch die gesamte EZB-Rechtsprechung aus Karlsruhe der letzten Jahre, auch wenn es selbst im jüngsten Urteil nicht in konkreten Grenzziehungen, sondern vor allem in einem skeptischen Grundraunen kulminiert.

Aus ökonomischer Sicht fällt es sehr schwer, der Argumentation des Gerichts zu folgen, dass sich Geldpolitik und Wirtschaftspolitik überhaupt klar abgrenzen lassen. Jede Entscheidung der Geldpolitik zieht weitreichende wirtschaftspolitische Implikationen nach sich. Insofern kann man argumentieren (und muss es aus ökonomischer Sicht wahrscheinlich sogar), dass durch die Entscheidung für die Europäische Währungsunion und damit für eine Geldpolitik in alleiniger Kompetenz der Europäischen Union bereits im Maastricht-Vertrag ein Schritt zum Föderalismus in der gesamten Wirtschafts- und Haushaltspolitik vollzogen wurde, der heute nicht mehr infrage gestellt werden kann.

Allerdings wird diese implizite „Föderalisierung“ als Konsequenz der Europäischen Währungsunion eben nur in Krisensituationen wirklich deutlich. Deshalb ist die Festlegung auf den Ausnahmeföderalismus als zentrale Funktionslogik der Währungsunion am Ende auch gar nicht überraschend. Mit der temporären Aktivierung föderaler Wirtschafts- und Haushaltsinstrumente umgeht die Politik die Antwort auf die Frage, ob die Wirtschafts- und Währungsunion eher eine dezentral strukturierte Wirtschaftsunion mit föderalen geldpolitischen Elementen ist – oder eine föderale Währungsunion mit dezentral ausgestalteten wirtschaftspolitischen Elementen. Solange dies so ist, bilden sich keine permanenten starken föderalen Institutionen. Und solange wird logisch die Hauptlast bei der einzigen existierenden föderalen Institution abgeladen – der Europäischen Zentralbank. Gerade die schärfsten Kritiker einer weiteren Föderalisierung kritisieren dies häufig ebenso scharf. Das passt nicht zusammen.

In welche Richtung werden sich Euroraum und Europäische Union also in den nächsten Jahren und Jahrzehnten bewegen? Es stehen dabei weiter drei grundsätzliche Optionen im Raum: Die erste wäre ein Weiter-So im Modus Ausnahmeföderalismus. Damit setzte sich der Euroraum allerdings der permanenten Gefahr aus, Krisen unterhalb der Auslösungsschwelle der föderalen Instrumente zu häufig und zu lange zu ignorieren, was letztlich zu nicht mehr umkehrbaren Divergenzen im Euroraum führen könnte. Gleichzeitig steht der Ausnahmeföderalismus immer auf institutionell und politisch tönernen Füßen, weil sein ganzes Wesen die Ausbildung starker gemeinsamer wirtschafspolitischer Institutionen jenseits der EZB verhindert. Option 2 wäre ein Rückbau der wirtschaftspolitischen Integration hin zu einem Maß, in dem Ausnahmeföderalismus nicht mehr notwendig ist. Hier sprechen wir allerdings nicht nur über eine Abwicklung der gemeinsamen Währung, sondern auch über einen weitgehenden Abbau der Integration über den Binnenmarkt. Option 3 schließlich wäre eine neue wirtschaftspolitische Verfasstheit für die EU und den Euroraum, der bestehende Interdependenzen endlich so anerkennt, dass starke gemeinsame Institutionen nicht mehr Ausnahme- sondern Regelfall werden. Die Konferenz zur Zukunft Europas sollte hier als Anlass genutzt werden, diese Grundsatzfrage anzugehen. Frankreich und Deutschland stünde es gut an, hier dem großen Ausnahmevorschlag einen großen Vorschlag für die Zukunft folgen zu lassen und die Debatte zu eröffnen.

## Schlussbemerkungen

Die aufgezeigten Optionen machen deutlich, dass auch die starken Antworten der Europäischen Union auf die Pandemie keine wirklichen Antworten zur Zukunft der Wirtschafts- und Währungsunion sind. Der Ausnahmeföderalismus ist einerseits ein Zeichen der Stärke der Europäischen Union, weil immer davon ausgegangen werden kann, dass in Krisen eine quasi-föderale Antwort auf jede Art der Herausforderung gefunden wird. Andererseits wird dieses Vorgehen aber auch von einer großen und permanenten Instabilität begleitet, erstens, weil jedes krisenbedingte Abweichen vom eigentlich vereinbarten nicht-föderalen Grundzustand die Legitimationsgrundlage aufzuweichen droht, und zweitens, weil immer die Frage im Raum steht, ob der föderale Sprung auch in der nächsten Krise gelingen kann.

Die am Ende sehr starken Krisenantworten Europas auf die Pandemie zeigen, wie handlungsfähig die Europäische Union in Krisenzeiten sein kann. Doch ob dieser Ausnahmeföderalismus ein dauerhaftes Erfolgsmodell sein kann, ist offen.

Prof. Dr. Henrik Enderlein ist Präsident und Professor für Politische Ökonomie an der Hertie School in Berlin sowie Direktor des Jacques Delors Centre.

Lucas Guttenberg, MPP, ist stellvertretender Direktor des Jacques Delors Centre an der Hertie School.


Literatur

Bénassy-Quéré, A. et al. (2018), Reconciling risk sharing with market discipline: A constructive approach to euro area reform, CEPR discussion paper, 91.
Dawson, M. et al. (Hrsg.) (2015), Beyond the Crisis, Oxford University Press.
Deutsche Bundesbank (2020), Pandemic Emergency Purchase Programme, https://www.bundesbank.de/de/aufgaben/geldpolitik/geldpolitische-wertpapierankaeufe/pandemic-emergency-purchase-programme-pepp-830356 (9. Juni 2020).
Enderlein, H. et al. (2012), A road map towards fi scal union in Europe, Report of the „Tommaso Padoa-Schioppa Group“, Notre Europe, Studies and Reports, 92.
Enderlein, H., L. Guttenberg und J. Spiess (2013), Blueprint for a Cyclical Shock Insurance in the Euro Area, Notre Europe-Jacques Delors Institute Report and Studies, 100.
Enderlein H Letta E Repair and Prepare: Growth and the Euro after Brexit 2016 Paris Bertelsmann Stiftung, Jacques Delors Institut — Berlin and Jacques Delors Institute
Enderlein, H. und L. Guttenberg (2019), The Eurozone’s Institutions at 20, in K. Bernoth et al., Happy birthday? The Euro at 20, Study for the Committee on Economic and Monetary Affairs, Policy Department for Economic, Scientific and Quality of Life Policies, European Parliament, 2019.
Grund, S., L. Guttenberg und C. Odendahl (2020), Sharing the Fiscal Burden of the Crisis: A Pandemic Solidarity Instrument for the EU, Jacques Delors Centre, Bertelsmann Stiftung Policy Paper.
Guttenberg, L. und J. Hemker (2018), No Escape from Politics: Four Tests for a Successful Fiscal Instrument in the Euro Area, Jacques Delors Institute Berlin, Bertelsmann Stiftung Policy Paper.
